# Bridging gaps across levels of care in rehabilitation of patients with rheumatic and musculoskeletal diseases: Results from a stepped-wedge cluster randomized controlled trial

**DOI:** 10.1177/02692155231153341

**Published:** 2023-03-02

**Authors:** Gunnhild Berdal, Anne-Lene Sand-Svartrud, Anita Dyb Linge, Ann Margret Aasvold, Kjetil Tennebø, Siv G Eppeland, Anne Sirnes Hagland, Guro Ohldieck-Fredheim, Helene Lindtvedt Valaas, Ingvild Bø, Åse Klokkeide, Joseph Sexton, Maryam Azimi, Turid N Dager, Ingvild Kjeken

**Affiliations:** 1 Norwegian National Advisory Unit on Rehabilitation in Rheumatology, Center for treatment of Rheumatic and Musculoskeletal Diseases (REMEDY), 11316 Diakonhjemmet Hospital, Oslo, Norway; 2 574813 Muritunet Rehabilitation Centre, Valldal, Ålesund, Norway; 3 Meråker Rehabilitation Centre, Meråker, Norway; 4 158956 Valnesfjord Health Sports Centre, Valnesfjord, Norway; 5 Department of Physiotherapy, Sørlandet Hospital, Arendal, Norway; 6 Haugesund Hospital for Rheumatic Diseases, Haugesund, Norway; 7 Vikersund Rehabilitation Centre, Vikersund, Norway; 8 Department of Rehabilitation, Hospital for Rheumatic Diseases, Lillehammer, Norway; 9 Rehabilitering Vest Rehabilitation Centre, Haugesund, Norway; 10 REMEDY Patient Advisory Board, Diakonhjemmet Hospital, Oslo, Norway

**Keywords:** Rheumatic and musculoskeletal diseases, multidisciplinary rehabilitation, complex interventions, goal setting, supportive follow-up, motivational interviewing, patient-centered, health behavior change, stepped-wedge cluster randomized trial, quality of life

## Abstract

**Objective:**

To compare the effectiveness of a structured goal-setting and tailored follow-up rehabilitation intervention with existing rehabilitation in patients with rheumatic and musculoskeletal diseases.

**Design:**

A pragmatic stepped-wedge cluster randomized trial.

**Setting:**

Eight rehabilitation centers in secondary healthcare, Norway.

**Participants:**

A total of 374 adults with rheumatic and musculoskeletal diseases were included in either the experimental (168) or the control group (206).

**Interventions:**

A new rehabilitation intervention which comprised structured goal setting, action planning, motivational interviewing, digital self-monitoring of goal progress, and individual follow-up support after discharge according to patients’ needs and available resources in primary healthcare (the BRIDGE-intervention), was compared to usual care.

**Main measures:**

Patient-reported outcomes were collected electronically on admission and discharge from rehabilitation, and after 2, 7, and 12 months. The primary outcome was patients’ goal attainment measured by the Patient Specific Functional Scale (0–10, 10 best) at 7 months. Secondary outcome measures included physical function (30-s Sit-To-Stand test), health-related quality of life (EQ-5D-5L-index), and self-assessed health (EQ-VAS). The main statistical analyses were performed on an intention-to-treat basis using linear mixed models.

**Results:**

No significant treatment effects of the BRIDGE-intervention were found for either primary (Patient Specific Functional Scale mean difference 0.1 [95% CI: −0.5, 0.8], *p* = 0.70), or secondary outcomes 7 months after rehabilitation.

**Conclusion:**

The BRIDGE-intervention was not shown to be more effective than existing rehabilitation for patients with rheumatic and musculoskeletal diseases. There is still a need for more knowledge about factors that can improve the quality, continuity, and long-term health effects of rehabilitation for this patient group.

## Introduction

Rheumatic and musculoskeletal diseases are among the largest contributors to disability worldwide.^1^ The diseases affect individuals by reducing physical and psychosocial health and constitute a substantial societal burden, which is predicted to rise markedly.^2^

Multidisciplinary rehabilitation is often required^3^ in form of coordinated health interventions applied to optimize function and minimize disability, with the overall purpose to enable patients to live their lives in line with personal preferences, needs, and goals.^4,5^

There is solid evidence that multidisciplinary rehabilitation provides beneficial health effects by improving function and health-related quality of life,^6–8^ but the effects tend to be small and decline quickly.^9,10^ One explanation may be that patients need continued support over a longer period of time to be able to reach their rehabilitation goals and implement new, healthy habits in their daily life.^11,12^ A lack of efficient and systematic follow-up after rehabilitation discharge may be one factor contributing to the diminishing effects.^13^ Further, without good communication, coordination, and continuity of care and support, patients may experience fragmented and poorly integrated health services from multiple providers, which again may result in suboptimal outcomes.^14,15^

The need for improved quality, continuity, and care coordination within and across healthcare settings is acknowledged as a challenge to be prioritized^14^ both internationally^16^ and in Norway.^17^ This will involve creating relationships and conditions to support informed and coherent interactions between the patient and multiple rehabilitation providers, within and across different locations, and over prolonged periods of time.^14,15^ Further, patient involvement and shared decision-making in the planning and tailoring of interventions are considered crucial to improve patient motivation, quality of care, adherence to treatment, self-care, and health outcomes.^16–19^

To strengthen the quality and continuity of rehabilitation, we developed the BRIDGE-intervention, designed to act as a bridge between healthcare levels. Underpinned by theories of behavioral change and empirical evidence of clinical effects, the BRIDGE-intervention comprised five main components: structured goal setting,^20,21^ action- and coping planning,^21^ digital self-monitoring of progress,^22^ tailored telephone follow-up support,^13,23^ and use of motivational interviewing^24,25^ in goal setting and follow-up conversations. We hypothesized that the intervention would help patients achieve their rehabilitation goals and improve or prolong beneficial health outcomes. Accordingly, the aim of this study was to evaluate if the BRIDGE-intervention was more effective than usual care in improving goal attainment and health outcomes in patients with rheumatic and musculoskeletal diseases.

## Methods

### Study design and settings

The trial was developed in line with the Medical Research Council recommendations for the design and evaluation of complex interventions,^26^ and built on several foregoing studies in which knowledge and tools for improving quality and continuity in rehabilitation were produced.^9,11–13,27–32^ Following a pilot feasibility cohort study,^11^ we performed a pragmatic stepped-wedge cluster randomized trial^33^ between August 2017 and August 2019. The stepped-wedge design was selected to facilitate patient recruitment and the practical implementation of the intervention at multiple sites, and to protect against potential between-group contamination and disappointment effects commonly associated with a parallel roll-out while maintaining a robust methodology for scientific evaluations.

Eight Norwegian rehabilitation centers (clusters) in secondary care participated, comprising five rehabilitation institutions and three hospital rheumatology departments. The centers started simultaneously to include patients in the control condition (delivering current rehabilitation) before they one after another introduced the BRIDGE-intervention in a predefined, randomized order. Accordingly, the intervention was rolled out in sequences with an initial period in which all centers provided usual care (control), and a closing period in which all centers provided the BRIDGE-intervention to patients (Box 1). The study was registered in ClinicalTrials.gov (NCT03102814) prior to launch.

Box 1. The BRIDGE-trial.

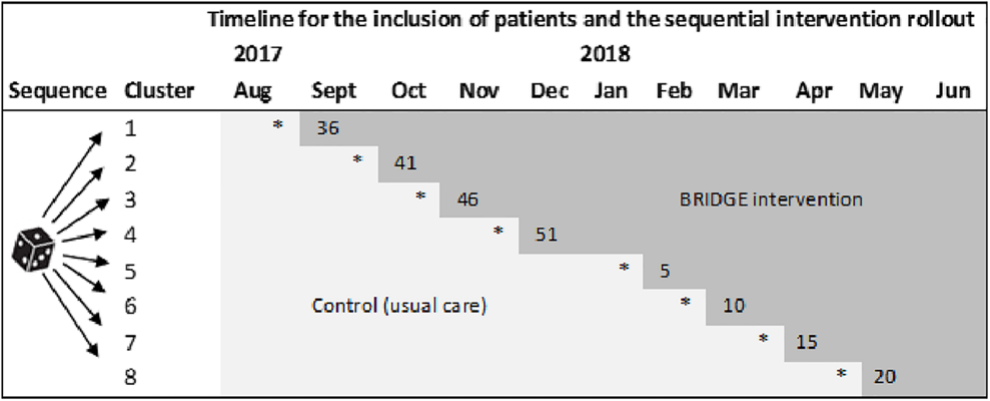

**The BRIDGE stepped-wedge cluster randomized trial design**. Patient enrolment started on 14 August 2017 and was closed on 30 June 2018. Light grey cells represent time periods for the inclusion of patients in the control group (usual care), and dark grey cells represent time periods for the inclusion of patients in the BRIDGE-intervention group. The asterisks indicate the timing of the research team's outreach visits for delivering the study materials and training of the intervention providers. Week numbers in which the clusters crossed over from the control condition to the intervention condition are provided in the initial dark grey cell for each cluster. The length of each step was 5 weeks (with an extension of 1 week for periods including national holidays). Patients recruited during the control condition stayed in the control group, and patients recruited in the intervention condition stayed in the intervention group. Individual patient-reported data were collected at 5 time points: admission and discharge, and 2, 7, and 12 months after admission to rehabilitation. The follow-up period continued throughout 2018 until the end of August 2019, implying a total trial duration of 106 weeks.
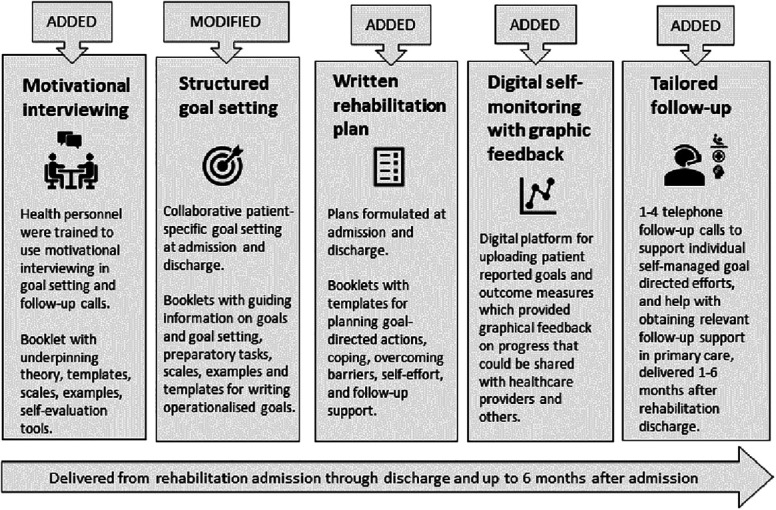
 **The BRIDGE-intervention** comprised five interacting components, which were partly added to, and partly provided to modify existing practice, supported by training by the study team and two guidance booklets, designed for patients and healthcare personnel, respectively. The intervention was designed to improve the quality and continuity of rehabilitation processes, with a focus on a high degree of patient involvement, follow-up support, and coordination of health services across levels of care.

### Participants

Patients were eligible for the study if aged ≥ 18 years; admitted to rehabilitation due to one of the following verified diagnoses: inflammatory rheumatic diseases, systemic connective tissue disorders, osteoarthritis, osteoporosis, fibromyalgia or chronic widespread pain, or non-specific low back, neck, or shoulder pain (persistent for > 3 months); able to read, understand and complete questionnaires in Norwegian; had access to a computer or equivalent device for digital data collection, and possessing a personal electronic credential for secure identification online. Exclusion criteria were cognitive impairment, severe psychiatric disorder(s), and fracture(s). Patients were screened for eligibility and recruited on admission to rehabilitation stay by local project coordinators at each participating center.

### Control and experimental interventions

Medical treatment as usual was provided to both groups. The control intervention consisted of the rehabilitation provided at the participating centers at the study start, which varied slightly in structure and content. Six centers offered inpatient stays of 3–4 weeks duration, while two hospital departments offered shorter stays of 2 weeks, either as an inpatient (center 7) or outpatient (center 4) rehabilitation.^1^ Multidisciplinary rehabilitation was provided through teams consisting of at least four health professions. The content of the rehabilitation provided included combinations of group sessions, individual sessions, and self-led activities. Details of the control intervention have been previously described^34^ (Additional file 1, available at https://doi.org/10.1186/s12913-021-06164-2). 

The BRIDGE-intervention was added to already existing rehabilitation and tailored to individual patients. The intervention was directed at improving the routines and communication skills of health professionals in secondary healthcare as regard goal setting, action planning, and psychological support, and at engaging and motivating patients to take an active role in pursuing their rehabilitation goals over time. Further, the intervention included telephone follow-up and help with connecting patients with required health- and social services, or other types of support, in their municipality of residence after rehabilitation discharge. Further, digital graphics based on self-reported outcomes were made available to the BRIDGE-intervention group (but not the control group), providing patient feedback on individual progression.

To facilitate the implementation of the BRIDGE-intervention, two booklets were designed to support health professionals and patients, respectively, with suggestions and templates for structured communication about, and registration of goals, action plans, and follow-up, along with information, instructions, communication tools, and illustrative examples. A preparatory reflection task (“The shoe”) and a YouTube tutorial on rehabilitation goals were made available to the patients before goal-setting meetings with healthcare personnel. The study materials are available online at https://diakonhjemmetsykehus.no/nkrr/prosjekter/bridge-studien.

An overview of the BRIDGE-trial, with a description of how and when the BRIDGE-intervention was implemented in relation to the control condition and the patients’ rehabilitation course, is provided in Box 1. Details on the content of the intervention components and the behavioral change techniques applied, with connections to the presumed mechanisms of action^35^ are provided in Appendix Table 1*.*

### Training of the intervention providers and fidelity monitoring

Training of the intervention providers was organized via outreach visits to each center by the research team (ALSS, IK, TND) who gave lectures, arranged workshops, and delivered the study materials. To minimize potential contamination of the control condition, the visits were arranged approximately one week before each center shifted to the intervention condition. During the trial, the centers received supervisory support from the chief study coordinator (ALSS), and regular project meetings were held that addressed study logistics and implementation.

To ensure and monitor that the BRIDGE-intervention was delivered according to the study protocol, a fidelity check list was used by the health professionals at each rehabilitation center (Appendix Table 2). Additionally, the patients reported their needs for follow-up after rehabilitation discharge, whether plans were made to meet these needs, and whether they had received follow-up, or not, in their municipality of residence. A quality indicator set for the rehabilitation of patients with rheumatic and musculoskeletal diseases^30^ was used to monitor the quality of the rehabilitation provided throughout the trial.

### Data collection

Patient-reported data were collected by means of an electronical portal, using a system delivered by Checkware (www.checkware.com). Information on socio-demographics, disease, medication, and lifestyle factors was collected at inclusion. Patient motivation for pursuing their rehabilitation goals was self-recorded at discharge from rehabilitation. All patients completed a core set of instruments for measuring outcomes of rehabilitation in rheumatic and musculoskeletal diseases^29^ on five occasions: on admission and discharge from rehabilitation, and after 2, 7, and 12 months in their home settings.

### Outcome measures

The applied rehabilitation core set includes patient-reported outcome measures that cover different aspects of health and function.^29^ The primary outcome in the present trial was patients’ goal attainment 7 months after rehabilitation measured by the Patient Specific Functional Scale.^36^ Baseline for this particular outcome was set at discharge because goals may change during a rehabilitation stay.^32^ In this instrument, the patients identified 1–5 goals in terms of “important activities that you have difficulty performing*,* and that you think are relevant rehabilitation goals for you to proceed with after discharge.” The patients rated their current status regarding each goal on 11-point numeric rating scales ranging from 0 (unable to perform) to 10 (able to perform without problems).

Secondary outcome measures were physical function, health-related quality of life, and self-assessed health status. Physical function was measured by the 30-s Sit-To-Stand Test.^37^ This is a clinical field test in which a chair of standard height is used and the maximum number of full stands, with arms crossed across the chest, during 30 s is recorded. An instructional video that demonstrated the correct performance of the test accompanied the written patient instructions. Health-related quality of life was measured by the generic questionnaire EQ-5D-5L (EuroQol 5 Dimensions, www.euroqol.org),^38^ which covers five domains: mobility, self-care, usual activities, pain/discomfort, and anxiety/depression. Each domain is scored on a five-level scale ranging from 1 (no problems) to 5 (unable to/extreme problems). Patient responses were transformed into summary index scores by applying societal preference weights based on normative reference material from a UK population.^39^ EQ-5D-5L-index scores range from below zero to 1, where 1 denotes the maximum health-related quality of life. Patients’ self-assessed overall health status was recorded on the vertical EuroQol-visual analog scale, numbered from 0 to 100, with the lower endpoint labeled “The worst health you can imagine” and the upper labeled “The best health you can imagine.”^38^

The remaining seven outcome measures of the core set were used as tertiary outcomes:

Coping was measured by the Effective Musculoskeletal Consumer Scale (0–100, 100 is best), a 17-item questionnaire assessing knowledge, attitudes, and behaviors about self-management skills.^29^ Functioning in daily activities was measured by the 12-item Hannover Functional Ability Questionnaire (0–24, 0 is best).^29^ Social participation was measured by one question from the COOP/WONCA questionnaire addressing whether “your physical or mental health has limited your social activities or contact with others,” rated on a scale from 1 to 5, where 1 is best.^29^ Mental health was measured by the Hopkins Symptom Checklist-5 (0–4, 0 is best), a five-item questionnaire assessing mainly anxiety and depression, or degree of psychological distress.^29^ Pain and fatigue were measured on 11-point numeric rating scales ranging from 0 to 10, where 0 is best (no pain, no fatigue).^29^ The patients’ motivation to work purposefully to achieve their stated goals was also measured on an equivalent scale; 0–10, where 10 is best (maximum motivation).^29^

### Sample size calculations

Sample size calculations were performed based on the primary outcome using information from a prior study testing the rehabilitation core set.^29^ It was estimated that a sample size of 140 participants (70 in each group) was needed to detect a mean difference of 1 point in the Patient Specific Functional Scale scores between the two groups, with a 0.05 significance level and a power of 80%, assuming a standard deviation of 1.84 for the scale, an intra-cluster correlation ρ (rho) < 0.005, approximately equal cluster sizes, and a dropout rate of 25%.

### Randomization and blinding

The cluster-level randomization was performed using a computer-generated list of random numbers. The eight clusters were given a number between 1 and 8 before they were randomly allocated to one of eight sequences (Box 1). The sequential intervention rollout plan was presented to the clusters at the study start, and accordingly, the intervention providers were not blinded to group allocation.

For patients, group allocation was determined by their admission dates, and whether the pertaining clusters were in the control- or intervention phase on that date. All patients were given identical study information on admission before consenting to participate, but to avoid unblinding^40^ disparate verbal information was given, by leaving out the particulars of the experimental intervention to potential participants in the control group. Hence, the patients knew they were trial participants and which treatment they were expected to receive, but they may have been blinded to which study group they belonged. Consequently, the patient-reported outcome assessments may also have been performed blinded to allocation status.

### Statistical analyses

Descriptive statistics were used to summarize and compare the baseline characteristics of the patients in the control- and intervention groups. The main statistical analysis for estimating treatment effects was performed with an intention-to-treat approach using all available data and a 3-level linear mixed model with random intercepts for the cluster (center) and patient (repeated measures), and with treatment group, baseline values, seasonal and calendar time treated as covariates. This method provided the estimated mean outcome level at each measurement time point, and the results of the group comparisons were reported as mean differences [intervention minus control] with 95% confidence intervals (CIs). Dropout analyses were performed to determine the response rate and attrition, and to explore the characteristics of completers and dropouts. The significance level was set at 0.05. Analyses were conducted in Stata IC16.

Several sensitivity analyses were conducted to investigate the robustness of our findings and the influence of missing data. These included within-cluster comparisons of the control- and intervention group, unadjusted intention-to-treat-analyses of the total sample using all available data, and rigorous per-protocol analyses. The per-protocol population was defined based on information from the fidelity checklist combined with patient-reported data on whether follow-up was needed and planned at discharge, and received within 7 months after rehabilitation (Appendix Table 2). To be included in the per-protocol analyses, a patient was required to fulfill 16 out of 19 protocol components, in addition to having contributed with data on the primary outcome.

Additionally, descriptive statistics were used to examine which components of the BRIDGE-intervention were received or not (yes/no) in the sub-group of patients who did not receive the BRIDGE-intervention as intended (the non-per protocol population). Further, we compared the control- and intervention group (total sample) with respect to patient-reported received follow-up (yes/no) in primary healthcare at 2 and 7 months after rehabilitation by using Pearson's Chi-square test.

### Patient and public involvement

Two patient research partners who participated as members of the project steering committee provided feedback on the project materials and the study's design-, conduct, and development. The steering committee further comprised one healthcare professional from each participating rehabilitation center who actively contributed with advice to better adapt the study activities to local conditions^26^ and to interpret the findings. Furthermore, two study-independent representatives from primary healthcare also provided advice, together with a representative from a tertiary healthcare rehabilitation unit, and an international expert in rehabilitation based in the Netherlands.

### Ethics

The study was approved by the Norwegian Regional Committee for Medical Research Ethics (REK South-East, 2017/665), and conducted in accordance with the Helsinki Declaration and the ICMJ Recommendations for the Protection of Research Participants. All study participants provided written, informed consent before enrolment.

## Results

### Participant flow

**Figure 1.**
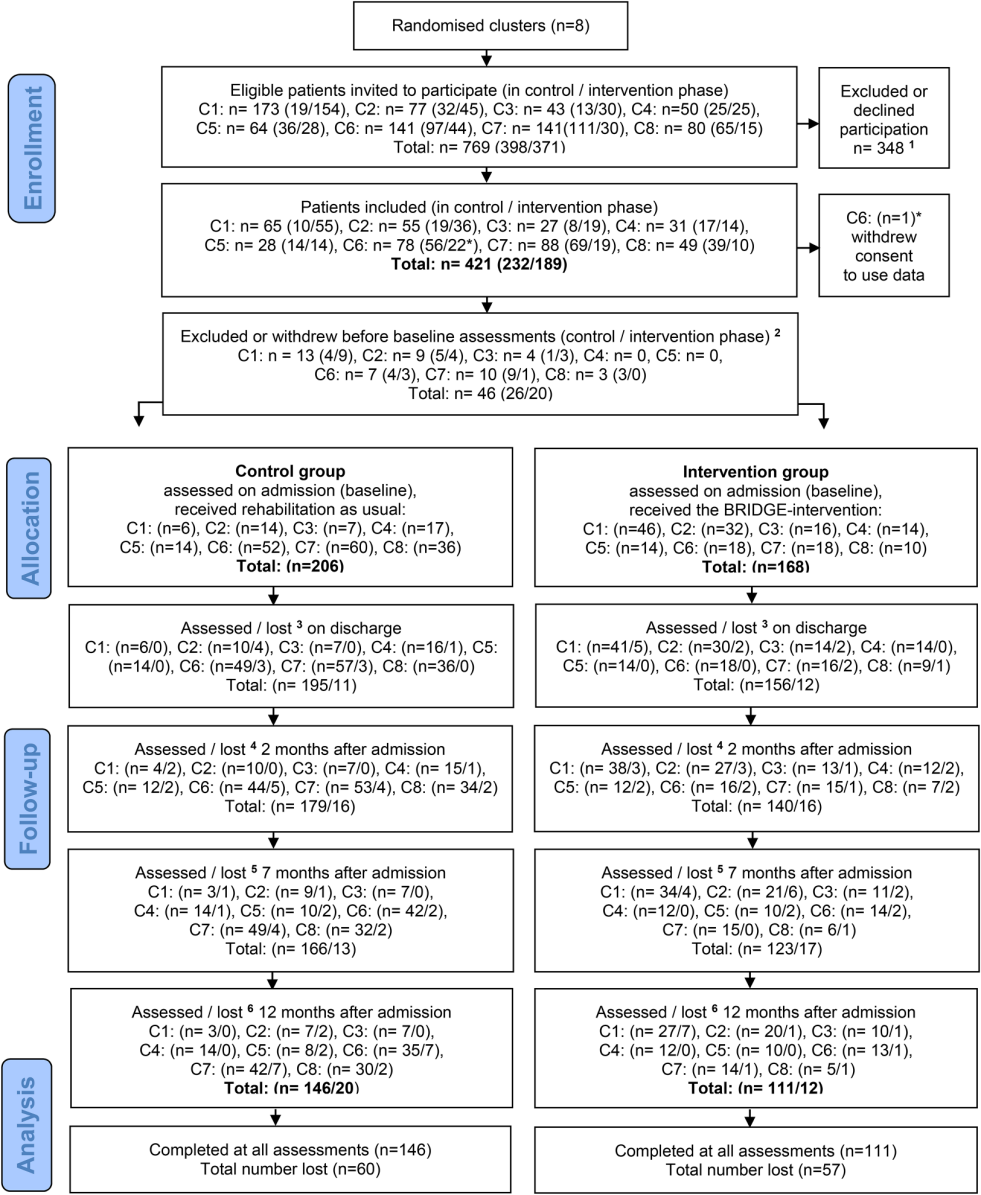
CONSORT flow diagram of patients.

Out of 769 eligible patients invited to participate, 421 consented. Of these, 46 withdrew or were excluded before baseline. A total of 374 patients (206/168 in the control/intervention group) completed the baseline assessments and were included in the analyses. Figure 1 depicts the flow of participants throughout the course of the study. The various stated reasons for dropout and exclusion are described for each group in Appendix Table 3.

C1–C8: clusters (center) 1–8.

^1–6^See Appendix Table 3 for details on reasons for refusal, withdrawal, and exclusion.

Dropout analyses comparing baseline characteristics of completers (*n* = 245) and non-completers (*n* = 129) of the primary outcome at 7 months follow-up showed that non-completers were more likely to be from the intervention group, were significantly younger, smokers had shorter disease duration, and more of them (27% vs. 16%) were diagnosed with fibromyalgia syndrome, compared to completers. At 12 months follow-up, this pattern was repeated, but with slightly smaller differences (data not shown).

### Baseline characteristics

The baseline characteristics of the included patients are shown in Table 1. The majority were middle-aged, married, women, with longstanding musculoskeletal disease, generally active, slightly overweight, and recipients of social security benefits, with relatively low levels of education. More than 70% used analgesics regularly, and approximately 20% used biological medicines. The treatment groups were mostly well-balanced with respect to baseline characteristics, however, some differences in the distribution of diagnoses were seen. The intervention group included a greater proportion of patients with fibromyalgia syndrome, while more patients with the inflammatory rheumatic disease were included in the control group. Further, the patients in the intervention group were significantly younger, with shorter disease duration compared to the control group.

**Table 1.** Baseline characteristics of the included patients (n = 374).

	Control group (n = 206)	Intervention group (n = 168)	*p*-value
Age, years, mean (min, max)	53 (21, 83)	49 (18, 77)	0.004^1^
Gender, female, n (%)	152 (74)	131 (78)	0.348^2^
Diagnosis, n (%)			
Inflammatory rheumatic disease^a^	148 (72)	90 (54)	< 0.001^2^
Osteoarthritis	8 (4)	5 (3)	
Connective tissue disease^b^	14 (7)	6 (4)	
Fibromyalgia syndrome, chronic widespread pain	21 (10)	55 (33)	
Unspecific neck-, shoulder- and low back pain	15 (7)	12 (7)	
Disease duration, years, median (min, max)	17 (1, 67)	13 (0, 68)	0.011^3^
Comorbidities, n, median (min, max)	25 (0, 9)	3 (0, 9)	0.379^3^
Medication usage			
Nonsteroidal anti-inflammatory drugs, n (%)	81 (42)	79 (53)	0.060^2^
Disease-modifying ant-rheumatic drugs (DMARDs), n (%)	70 (37)	52 (35)	0.705^2^
TNF-inhibitors, Biosimilars, JAK-inhibitors n (%)	44 (23)	26 (17)	0.196^2^
Analgesics, n (%)	136 (71)	108 (72)	0.872^2^
Other drugs, n (%)	138 (72)	113 (75)	0.522^2^
BMI (kg/m^2^), median (min, max)	28 (17, 66)	28 (17, 50)	0.654^3^
Smokers, n (%)	58 (28)	38 (23)	0.265^2^
Snuff users, n (%)	19 (10)	15 (9)	0.950^2^
Education > 12 years, n (%)	83 (41)	70 (43)	0.699^2^
Paid work, n (%)	86 (42)	73 (45)	0.594^2^
Recipients of social security benefits, n (%)	144 (81)	127 (86)	0.222^2^
Living with partner, n (%)	143 (70)	112 (68)	0.762^2^
Physical exercise ≥ 1 per week, n (%)	129 (63)	88 (54)	0.072^2^
General activity ≥ 1 per week, n (%)	152 (74)	109 (67)	0.125^2^

a Spondyloarthritis, psoriatic arthritis, rheumatoid arthritis, and juvenile rheumatoid arthritis.

b Systemic lupus erythematosus, Sjögren syndrome, polymyalgia rheumatic, and mixed connective tissue disease. Disease duration (symptom debut) and comorbidities are self-reported. DMARDS include corticosteroids. TNF: tumor necrosis factor; JAK: Janus Kinase; BMI: body mass index (bodyweight/height^2^). Physical exercise: increased heart rate and breathing for 30 min or longer. General activity: social or cultural activities, hobbies, and work.

1 Independent samples *t*-test.

2 Pearson's Chi-square test.

3 Mann-Whitney *U*-test.

### Effect outcomes

**Figure 2.**
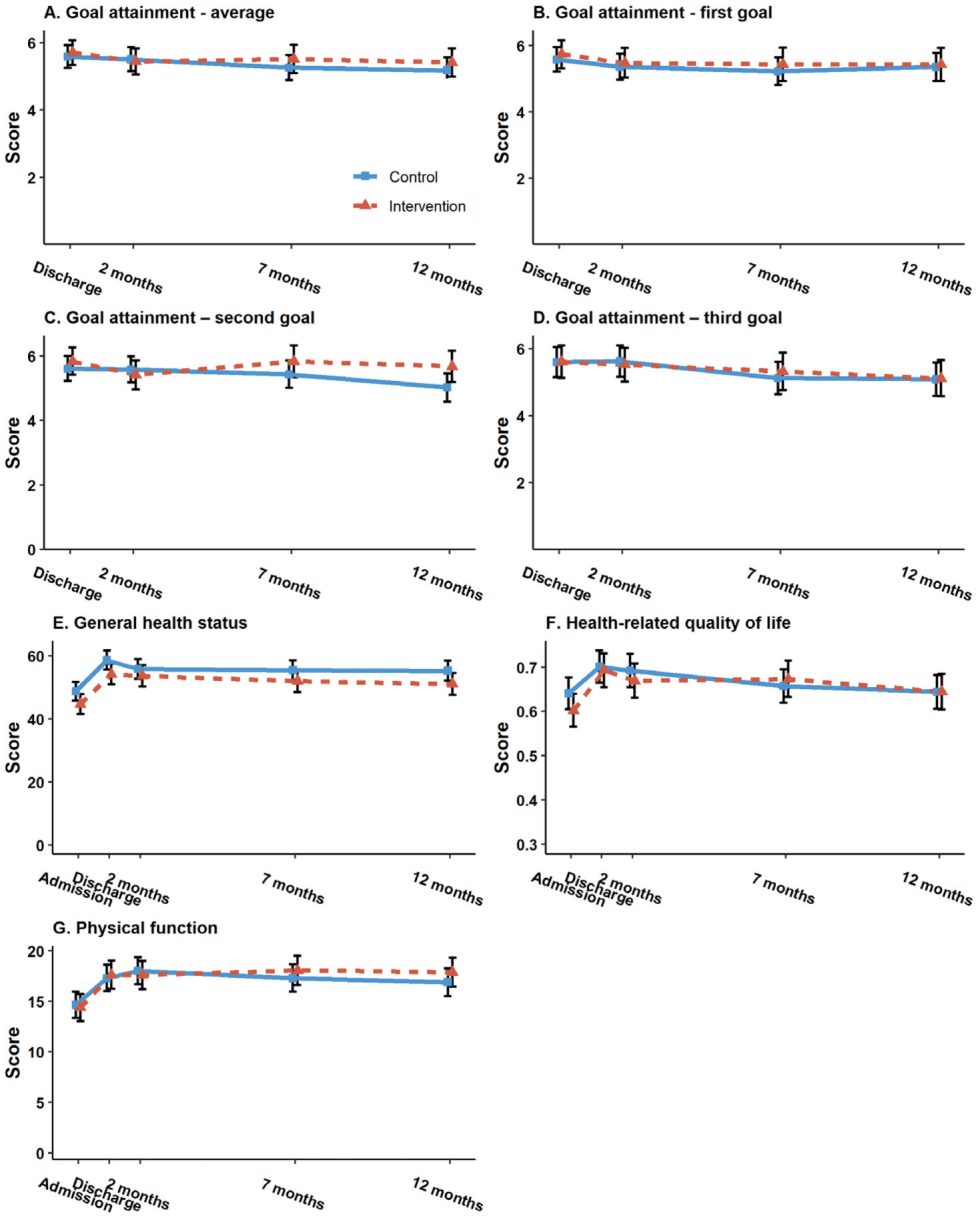
Goal attainment measured with the Patient-Specific Functional Scale (0–10, 10 best) indicated by the ability to perform goal (activity) at 4 time points: discharge from rehabilitation (baseline for this particular outcome) and after 2, 7, and 12 months. (A) The average of the first three goals set by the patients was measured over time, while (B to D) the results separately for the first, second, and third goalss, respectively. (E) General health status was measured with the EuroQol Visual Analog Scale (0–100), (F) health-related quality of life was measured with the 5-level EuroQol 5-Dimensions index (0-1), and (G) physical function was measured with the 30-s Sit-to-Stand test at 5 time points: on admission and discharge from rehabilitation, and after 2, 7, and 12 months. Vertical lines indicate the estimated mean values (center) with 95% confidence intervals, and horizontal lines show the fluctuating mean values from baseline to 12 months. Dotted line = intervention group. Unbroken line = control group.

The results from the main intention-to-treat analyses are presented in Table 2. No significant treatment effects were found for primary or secondary outcomes. For the primary outcome; goal attainment, a negligible between-group difference of 0.1 [95% CI −0.5, 0.8], *p* = 0.70 was found, with a confidence interval indicating at most modest effects. Similar results were found for the secondary outcomes; physical function (mean difference 0.9 [95% CI: – 0.4, 2.2], *p* = 0.18), health-related quality of life (mean difference 0.0 [95% CI: −0.0, 0.0], *p* = 0.99), and self-assessed health (mean difference −0.1 [95% CI: −4.1, 3.9], *p* = 0.98) at 7 months follow-up. However, for the tertiary outcome functioning in daily activities, a significant result in favor of the intervention group was found (mean difference −1.7 [95% CI −2.7, −0.7], *p* = 0.001), which remained significant after a Bonferroni Correction. However, the effect was not maintained at 12 months. For both groups, the mean outcome scores remained fairly stable throughout all measurement time points from discharge to 12 months follow-up. Figure 2 shows the development over time in the primary and secondary outcome scores from baseline to the 12 months follow-up, by treatment arm.

**Table 2.** Mean difference scores for treatment effects.^a^

Outcomes	Control group Mean (SD)	Intervention group Mean (SD)	Treatment effect Mean difference (95% CI)	p*
***Primary outcome***				
**Patient-Specific Functional Scale mean ability score for goals 1, 2, and 3** (0–10, 10 is best)^b^				
Discharge^(baseline)^	5.6 (2.0)	5.7 (2.3)	0.1 (−0.4, 0.5)^c^	
2 months	5.6 (1.9)	5.4 (2.1)	−0.2 (−0.7, 0.4)	
7 months^e^	5.4 (2.2)	5.5 (2.0)	0.1 (−0.5, 0.8)	0.703
12 months	5.3 (2.2)	5.4 (2.2)	0.2 (−0.5, 0.8)	

**Patient-Specific Functional Scale mean ability score for goal 1** (0–10, 10 is best)^b^				
Discharge^(baseline)^	5.6 (2.4)	5.7 (2.8)	0.1 (−0.4, 0.7)^c^	
2 months	5.4 (2.5)	5.5 (2.7)	−0.1 (−0.8, 0.7)	
7 months^e^	5.3 (2.7)	5.5 (2.8)	0.2 (−0.7, 1.0)	0.723
12 months	5.5 (2.6)	5.5 (2.8)	−0.1 (−0.9, 0.7)	

**Patient-Specific Functional Scale mean ability score for goal 2** (0–10, 10 is best)^b^				
Discharge^(baseline)^	5.6 (2.3)	5.8 (2.5)	0.2 (−0.3, 0.8)^c^	
2 months	5.7 (2.3)	5.4 (2.6)	0.0 (−0.7, 0.8)	
7 months^e^	5.5 (2.6)	5.7 (2.6)	0.4 (−0.4, 1.2)	0.352
12 months	5.2 (2.5)	5.6 (2.7)	0.8 (0.0, 1.6)	

**Patient-Specific Functional Scale mean ability score for goal 3** (0–10, 10 is best)^b^				
Discharge^(baseline)^	5.7 (2.4)	5.6 (2.7)	−0.1 (−0.6, 0.5)^c^	
2 months	5.8 (2.4)	5.5 (2.7)	−0.4 (−1.2, 0.3)	
7 months^e^	5.3 (2.6)	5.3 (2.6)	−0.1 (−0.9, 0.7)	0.772
12 months	5.3 (2.7)	5.1 (2.9)	−0.3 (−1.1, 0.5)	

***Secondary outcomes***				
**The 30 s Sit to Stand Test** (higher number better)^b^				
Admission ^(baseline)^	14.5 (5.6)	14.2 (4.8)	−0.3 (−1.8, 1.2)^c^	
Discharge	17.2 (7.0)	17.6 (6.6)	0.2 (−0.9, 1.3)	
2 months	17.9 (7.5)	17.6 (7.6)	−0.7 (−1.8, 0.5)	
7 months^e^	17.3 (7.5)	17.9 (7.1)	0.9 (−0.4, 2.2)	0.183
12 months	17.4 (7.3)	17.9 (7.5)	1.0 (−0.3, 2.2)	

**5-level EuroQol 5-Dimensions index** (index value 0–1, 1 is best)^b^				
Admission ^(baseline)^	0.6 (0.2)	0.6 (0.2)	−0.0 (−0.1, 0.0)^c^	
Discharge	0.7 (0.2)	0.7 (0.2)	−0.0 (−0.0, 0.0)	
2 months	0.7 (0.2)	0.7 (0.2)	−0.0 (−0.1, 0.0)	
7 months^e^	0.7 (0.2)	0.7 (0.2)	0.0 (−0.0, 0.0)	0.985
12 months	0.7 (0.2)	0.6 (0.2)	0.0 (−0.0, 0.0)	

**EuroQol Visual Analog Scale** (0–100, 100 is best)^b^				
Admission ^(baseline)^	48.5 (18.6)	44.5 (16.2)	−4.1 (−8.0, −0.2)^c^	
Discharge	58.2 (17.8)	53.9 (18.5)	−1.3 (−4.6, 2.0)	
2 months	55.9 (18.2)	54.1 (18.2)	0.1 (−3.4, 3.7)	
7 months^e^	56.2 (19.6)	51.8 (18.0)	−0.1 (−4.1, 3.9)	0.977
12 months	56.4 (19.9)	51.0 (18.7)	−0.4 (−4.2, 3.3)	

***Tertiary outcomes***				
**Pain, Numeric Rating Scale** (0–10, 0 is best)^d^				
Admission ^(baseline)^	5.7 (2.0)	6.1 (2.0)	0.3 (−0.1, 0.7)^c^	
Discharge	4.9 (2.1)	5.4 (2.1)	0.2 (−0.1, 0.6)	
2 months	5.2 (2.1)	5.6 (1.9)	0.2 (−0.3, 0.6)	
7 months	5.2 (2.3)	5.3 (1.8)	−0.4 (−0.9, 0.1)	0.091
12 months	5.2 (2.2)	5.8 (2.1)	0.3 (−0.1, 0.7)	

**Fatigue, Numeric Rating Scale** (0–10, 0 is best)^d^				
Admission ^(baseline)^	6.0 (2.8)	6.5 (2.7)	0.5 (−0.1, 1.0) ^c^	
Discharge	4.9 (2.7)	5.6 (2.8)	0.4 (−0.1, 0.8)	
2 months	5.3 (2.6)	6.0 (2.6)	0.6 (0.1, 1.1)	
7 months	5.5 (2.8)	5.9 (2.6)	0.1 (−0.5, 0.6)	0.795
12 months	5.5 (2.8)	5.9 (2.7)	0.1 (−0.4, 0.7)	

**Hannover Functioning Questionnaire** (0–24, 0 is best) ^d^				
Admission ^(baseline)^	10.0 (4.6)	10.0 (4.5)	0.1 (−1.0, 1.2) ^c^	
Discharge	9.4 (4.6)	9.2 (4.8)	−0.2 (−1.1, 0.6)	
2 months	9.2 (5.2)	8.6 (5.4)	−0.9 (−1.7, 0.0)	
7 months	9.1 (5.6)	8.2 (6.0)	−1.7 (−2.7, −0.7)	0.001
12 months	8.7 (5.5)	9.4 (5.7)	−0.1 (−1.0, 0.9)	

**Effective Musculoskeletal Consumer Scale** (0–100, 100 is best)^b^				
Admission ^(baseline)^	65.2 (14.5)	60.0 (14.8)	−3.5 (−6.7, −0.3) ^c^	
Discharge	67.2 (14.0)	63.5 (14.3)	−0.6 (−3.0, 1.7)	
2 months	68.3 (14.7)	64.0 (15.7)	−1.1 (−3.6, 1.4)	
7 months	67.8 (14.6)	64.5 (14.9)	−0.6 (−3.4, 2.2)	0.668
12 months	67.5 (14.9)	65.3 (15.1)	1.7 (−0.9, 4.3)	

**Hopkins Symptom checklist** (0–4, 0 is best)^d^				
Admission ^(baseline)^	1.1 (0.9)	1.3 (1.1)	0.2 (−0.0, 0.3) ^c^	
Discharge	0.8 (0.7)	1.0 (0.8)	0.0 (−0.1, 0.2)	
2 months	0.8 (0.7)	1.0 (0.9)	0.1 (−0.0, 0.2)	
7 months	0.9 (0.8)	1.0 (0.9)	0.0 (−0.2, 0.2)	0.963
12 months	1.0 (0.9)	1.0 (1.0)	−0.1 (−0.3, 0.0)	

**Social Participation subscale of COOP/WONCA** (1–5, 1 is best)^d^				
Admission ^(baseline)^	2.6 (1.2)	2.9 (1.2)	0.3 (0.1, 0.5) ^c^	
Discharge	2.2 (1.0)	2.4 (1.1)	0.1 (−0.1, 0.3)	
2 months	2.2 (1.0)	2.5 (1.2)	0.3 (0.1, 0.5)	
7 months	2.3 (1.1)	2.5 (1.1)	0.1 (−0.2, 0.3)	0.573
12 months	2.4 (1.2)	2.7 (1.3)	0.3 (0.0, 0.5)	

**Patient-Specific Functional Scale mean motivation score for goal 1** (0–10, 10 is best)				
Discharge	8.2 (2.0)	8.5 (1.7)	0.5 (0.1, 1.0)	

**Patient-Specific Functional Scale mean motivation score for goal 2** (0–10, 10 is best)				
Discharge	8.4 (1.7)	8.4 (1.8)	0.1 (−0.3, 0.6)	

**Patient-Specific Functional Scale mean motivation score for goal 3** (0–10, 10 is best)				
Discharge	8.2 (2.0)	8.4 (1.9)	0.4 (−0.1, 0.9)	

a Estimated with linear mixed models. SD: standard deviation; CI: confidence interval. *Significant at *p* < 0.05.

b Adjusted for baseline values, seasonal effects, calendar time, and multilevel sampling: positive values favor the intervention group.

c Unadjusted baseline values.

d Adjusted for baseline values, seasonal effects, calendar time, and multilevel sampling: negative values favor the intervention group.

e Primary end-point.

COOP/WONCA: The Dartmouth Primary Care Cooperative Information Project/World Organization of National Colleges, Academies, and Academic Associations of General Practice/Family Physicians questionnaire. Numbers analyzed (n_a_) in the control/intervention group for the Patient Specific Functional Scale (PSFS) discharge: n_a_ = 193/152, PSFS 2 months: n_a_ = 167/130, PSFS 7 months: n_a_ = 146/99, and PSFS 12 months: n_a_ = 136/102.

The per-protocol analyses produced similar results to the intention-to-treat-analyses (adjusted/unadjusted) with respect to group differences and *p*-values. However, only 31% (n = 52/168) of the patients in the intervention group were included in the per-protocol analyses. Of those excluded (*n* = 116) from the per-protocol analyses, 14 lacked checklist information. Of the remaining (n = 102) nearly all received the BRIDGE-intervention components delivered in secondary healthcare, but most (*n* = 99) reported not to have received required follow-up (health- and social services) in primary care after discharge.

The results from the within-cluster comparisons of the control- and intervention groups (data not shown) also produced similar results with group differences near zero, with one exception (center 2) demonstrating a moderate positive effect of the BRIDGE-intervention, compared to the control condition (mean difference 2.7 [95% CI 0.3, 5.1]).

### Follow-up received

Explorative comparisons of the control- and intervention group with respect to patient-reported received follow-up in primary healthcare showed few significant differences, except that the control group received more follow-up from nurses at medical doctors’ offices within 2 months after rehabilitation, and that the intervention group received more follow-up from the social security office within 7 months after rehabilitation. As concerns the healthcare services most frequently used by the patients, the groups were relatively similar (Table 3).

**Table 3.** Patient-reported received follow-up (use of health- and social services) in primary healthcare or other sectors^a^ after rehabilitation in secondary healthcare.

	Control group (n = 206)	Intervention group (n = 168)	*p*-value
**Yes, within 2 months after rehabilitation**			
General practitioner, n consultation's^b^ (%)	104 (65)	90 (69)	0.505^1^
Specialized medical doctor, n consultation's^b^ (%)	54 (35)	42 (33)	0.821^1^
**Yes, within 2 months after rehabilitation**			
Physical therapist (yes/no), n (%)	80 (39)	63 (38)	0.792^1^
Chiropractor (yes/no), n (%)	9 (4)	5 (3)	0.480^1^
Occupational therapist (yes/no), n (%)	9 (4)	7 (4)	0.923^1^
Home care nurse service (yes/no), n (%)	1 (0.5)	1 (0.6)	0.885^1^
Nurse at medical doctor's office (yes/no), n (%)	22 (11)	7 (4)	0.019^1^*
Psychiatric nurse (yes/no), n (%)	1 (0.5)	3 (2)	0.224^1^
Psychologist (yes/no), n (%)	4 (2)	9 (5)	0.073^1^
Multidisciplinary rehabilitation (yes/no), n (%)	1 (0.5)	1 (0.6)	0.885^1^
Health life center (yes/no), n (%)	6 (3)	4 (2)	0.751^1^
Norwegian labor and welfare administration (yes/no), n (%)	18 (9)	21 (13)	0.236^1^
Other healthcare services (yes/no), n (%)	20 (10)	15 (9)	0.797^1^
Alternative medicine (e.g., acupuncture, homeopathy), n (%)	5 (3)	9 (7)	0.129^1^
Need for primary healthcare services not met (yes/no), n (%)	12 (7)	11 (8)	0.769^1^
**Yes, within 7 months after rehabilitation**			
General practitioner, n consultation's^b^ (%)	127 (85)	86 (86)	0.886^1^
Specialized medical doctor, n consultation's^b^ (%)	84 (57)	65 (66)	0.149^1^
**Yes, within 7 months after rehabilitation**			
Physical therapist (yes/no), n (%)	83 (40)	60 (36)	0.365^1^
Chiropractor (yes/no), n (%)	12 (6)	9 (5)	0.845^1^
Occupational therapist (yes/no), n (%)	15 (7)	7 (4)	0.203^1^
Home care nurse service (yes/no), n (%)	0 (0)	1 (0.5)	0.268^1^
Nurse at medical doctor's office (yes/no), n (%)	18 (9)	9 (5)	0.209^1^
Psychiatric nurse (yes/no), n (%)	5 (2)	4 (2)	0.977^1^
Psychologist (yes/no), n (%)	7 (3)	12 (7)	0.101^1^
Multidisciplinary rehabilitation (yes/no), n (%)	4 (2)	3 (2)	0.912^1^
Health life center (yes/no), n (%)	8 (4)	4 (2)	0.412^1^
Norwegian labor and welfare administration (yes/no), n (%)	21 (10)	31 (18)	0.022^1^*
Other healthcare services (yes/no), n (%)	11 (5)	9 (5)	0.994^1^
Alternative medicine (e.g., acupuncture, homeopathy), n (%)	10 (8)	11 (12)	0.249^1^
Need for primary healthcare services not met (yes/no), n (%)	7 (5)	10 (10)	0.103^1^

^a^ The Norwegian labor and welfare administration is organized on the state level, but its services are integrated and provided in the local municipalities. Specialized medical doctors are organized in secondary healthcare, but can provide health services in local municipalities. Alternative medicine resides in the private sector, outside of public health services.

^b^ Number of consultations since last registration summarized across patients.

*Significant at *p* < 0.05.

^1^ Pearson's Chi-square test.

### Missing data

A high proportion of missing data was exposed. Missing data occurred both as consequence of attrition and incomplete completion of questionnaires. For the primary outcome, the proportions of missing data were 29% in the control- and 41% in the intervention group at 7 months follow-up (Table 2, numbers analyzed provided in the subtext). Similar proportions of missing data were also found for the other outcome measures at 7 months. Attrition was 19% (40/206) in the control group and 27% (45/168) in the intervention group at 7 months (Figure 1), which means that 10% and 14% of the missing data in the control- and intervention group, respectively, occurred due to inadequate completion of questionnaires by patients who stayed in the study at this point.

## Discussion

This study aimed to evaluate the effectiveness of providing a multicomponent health behavior change intervention (the BRIDGE-intervention) as an adjunct to usual care in the rehabilitation of patients with rheumatic and musculoskeletal diseases. The results showed no significant treatment effects of the BRIDGE-intervention on either goal attainment, physical function, health-related quality of life, or self-assessed health. For both groups, the mean primary and secondary outcome scores remained fairly stable from discharge to 12 months follow-up, indicating small, but lasting effects of the rehabilitation provided to both groups.

Our findings differ from the findings of a recent systematic review of reviews summarizing the evidence base for psychological interventions in patients with rheumatoid arthritis,^41^ concluding that intervention components such as goal setting, action planning, motivational interviewing, and supportive counseling, result in small to moderate improvements in biopsychosocial patient outcomes in addition to those achieved by the standard care, and that longer lasting interventions with a maintenance component appear particularly effective. They also deviate from those of two other systematic reviews^42,43^ reporting that combining many health behavior change techniques yield higher effects and increase the possibility of achieving health behavior change in patients with chronic musculoskeletal conditions.

One possible reason for the lack of effect in our study may be related to implementation difficulties. The BRIDGE-intervention was highly complex,^26^ comprising numerous interacting components, that were delivered by a variety of healthcare personnel, within a range of different real-life settings, thereby providing considerable room for variation in the delivery of the intervention. To optimize implementation, the BRIDGE-intervention was highly protocolized with detailed manuals to guide both health professionals and patients in adhering to the intervention procedures throughout the study. However, this may unintentionally have added to the complexity of both delivering and receiving the intervention.

This hypothesis is supported by the fidelity estimates, which showed that only 31% of the patients in the intervention group received the BRIDGE-intervention as intended, with follow-up after rehabilitation discharge as the most frequently lacking factor. Our findings thereby confirm other reports stating that care coordination across service levels still are among the weakest elements in the rehabilitation process.^11,14,44^

As the BRIDGE-intervention specifically targeted improvement of the transitions between levels of care, we expected that patients in the intervention group would receive more follow-up than the control group. However, we found that the groups received an approximately equivalent amount of follow-up, reported as a use of health- and social services after rehabilitation discharge. Variations in cluster-level delivery of the BRIDGE-intervention may potentially explain the observed variation in the primary outcome between clusters. The way institutions and individuals adopt and integrate new trial-related work into existing routines, is influenced by the degree to which the new work is perceived to be relevant and legitimate.^45^ Further, provider-level factors, such as experience, engagement, knowledge, and skills may influence the fidelity of delivery.^46^ Ultimately, the responses and actions of the individuals who received the BRIDGE-intervention must have influenced its effectiveness,^45^ as it depended on these individuals changing behavior in relation to set goals.

A strength of this study is the robust research design, which may have kept the included patients blinded to group affiliation and thus protected against disappointment effects and contaminant factors during the intervention roll-out, which strengthens the internal validity of the study. Further, the pragmatic orientation enabled evaluations in real-world settings, strengthening the external validity. Other methodological strengths are the use of the rehabilitation core set of outcomes for patients with rheumatic and musculoskeletal diseases,^29^ reflecting the patient-reported desired effects of rehabilitation on everyday functioning across several life domains, as well as the rigorous statistical analyses accounting for calendar time and data clustering.

The study has several limitations. The cluster randomized study design is prone to post-randomization selection bias,^40^ and there is a risk of performance bias as it was not possible to blind the intervention providers. Moreover, several reports stress that there might be a risk of both type I and type II errors associated with few clusters in stepped-wedge cluster randomized trials.^47,48^ Although the exact lower limit for a number of clusters in such trials is currently not known, the BRIDGE trial would probably have benefited from including more than eight clusters.

A substantial limitation is a large amount of missing data, which reduces the statistical power and the representativeness of the sample, and impacts on the validity and generalizability of the results. Another limitation is the lack of longitudinal data on patient motivation and self-managed goal-directed activity after rehabilitation discharge, which could have provided valuable information in the interpretation of the findings. Finally, the fact that missing data and attrition were highest in the experimental group compared to the control group, may also indicate that receiving in the BRIDGE-intervention was burdensome to some patients.

This study demonstrates that shortcomings still exist in rehabilitation transitions between levels of care. The multicomponent BRIDGE-intervention was not shown to be more effective than usual care in terms of improving goal attainment, physical function, health-related quality of life, and self-assessed health in patients with rheumatic and musculoskeletal diseases. However, the implementation of the intervention was not optimal, as the majority of patients did not receive the required follow-up in their municipality of residence after rehabilitation discharge. This finding will be further explored in an upcoming study. Further research should focus on factors that can raise treatment fidelity among health care providers, bridge gaps across levels of care, and thus improve the quality, continuity, and long-term health effects of rehabilitation for this patient group.

**Clinical messages**

- The BRIDGE-intervention did not lead to better goal attainment and clinical outcomes in the rehabilitation of patients with rheumatic and musculoskeletal diseases, compared with usual care.
- Further research is required to improve the continuity of rehabilitation across levels of care and the long-term health effects of rehabilitation for this patient group.

## Notes

1. These two centres correspond to clusters 4 and 7 in Figure 1 in the present paper, but to centres 2 and 6, respectively, in the link provided (Additional file 1).^34^

## Appendix

**Table A1.** Description of the BRIDGE-intervention with the behavior change techniques used and the presumed theoretical mechanisms of action according to The Behavior Change Technique Taxonomy* and The Theory and Techniques Tool.**

Component content and mode of delivery^Ω^		Behavior change technique	Mechanism of action
*1*	*Structured goal setting:* In collaboration with the multidisciplinary team, patients discussed and set from one to maximum of five rehabilitation goals during goal-setting meetings organized at admission and discharge from rehabilitation. The goal-setting process was structured according to the G-AP approach^1^ and included four stages: (i) goal negotiation and goal setting, (ii) action planning, coping planning and measurement of confidence to complete plan, (iii) action (carry out plan), and (iv) evaluation, feedback, and reorientation. The process was guided by detailed instructions in two booklets designed for patients and healthcare professionals, respectively. The booklets contained information about goals and goal setting, a reflection task (the shoe) designed to stimulate goal development, examples of goal statements, and templates with step-by-step instructions on how to operationalize goals according to the SMART^§^ approach, and with open fields for writing goals. A tutorial video on rehabilitation goal setting was developed particularly for the trial and made available on www.youtube.com^β^ for supplementary guidance. Once the goals were set, they were used as a basis for tailoring the rehabilitation interventions. To monitor progress, the goals were electronically recorded in the PSFS^¶^ by the patients and scored in terms of difficulty to perform on five occasions throughout the study period.	Goal setting (behavior) [1.1] Goal setting (outcome) [1.3] Behavioral contract [1.8] Commitment [1.9] Graded tasks [8.7] Mental rehearsal of successful performance [15.2] Review behavior goal(s) [1.5] Review of outcome goal(s) [1.7]	Intention^#^ Goals^#^ Beliefs about capabilities^¤^ Behavioral regulation ^¤^ Motivation^#^ Goals ^¤^ Intention ^¤^ Memory attention and decision process ^¤^ Skills^#^ Beliefs about capabilities^#^ Goals ^¤^ Motivation ^¤^ Values ^¤^ Goals^#^ Goals^#^
2	*Plans for self-management and follow-up:* Strategies for achieving individual rehabilitation goals and maintaining self-management and health-promoting behavior were discussed with the rehabilitation team and specified by the patient in written plans using predefined templates, including if-then plans by identifying potential risk situations, difficulties, and barriers, as well as sources of support. Specific examples of such plans were available, along with a scale (ranging from 0 to 10) on which the patients could rate their confidence to complete their plan. Before discharge, follow-up plans were developed by the multidisciplinary team in collaboration with the patients. These plans were tailored to the patients’ individual needs and available resources in their municipality of residence.	Action planning (including implementation intentions) [1.4] Problem-solving/coping planning [1.4] Discrepancy between current behavior and goal standard [1.6] Valued self-identity/ Self-affirmation [13.4]	Behavioral cueing^#^ Behavioral regulation ^¤^ Behavioral regulation^#^ Beliefs about capabilities^#^ Skill ^¤^ Environmental context and resources ^¤^ Goals^#^ Feedback processes^#^ Behavioral regulation ^¤^ Intention^¤^
3	*Digital self-monitoring with individualized feedback:* By using an electronic portal, patients completed a rehabilitation core set of instruments measuring their health and function five times over a one-year period. The results were visualized in a graphic report, enabling the patients to monitor their progress and share with family, friends, or healthcare personnel in primary health care.	Self-monitoring of the outcome of behavior [2.4] Social support (general) [3.1]	Behavioral regulation ^¤^ Social influence^#^ Social/professional role and identity ^¤^
4	*Tailored follow-up* One month after discharge, all patients received 1 mandatory telephone follow-up call from their primary contact at the rehabilitation center, providing support with goal progress and self-management, and addressing potential needs for help to establish contact with local health- and welfare services, or other types of support. The patients were further offered to receive up to 3 additional telephone calls (optional, based on individual needs) within a six-month period after rehabilitation discharge.	Review behavior goal(s) [1.5] Review of outcome goal(s) [1.7] Feedback on behavior [2.2] Other(s) monitoring with awareness [2.1 and 2.5] Social support (general) [3.1] Social support (practical) [3.2] Feedback on outcome(s) of behavior [2.2] Self-monitoring of behavior [2.3] Self-monitoring of outcomes of behavior [2.4]	Goals^#^ Goals^#^ Motivation^#^ Feedback processes^#^ Optimism ^¤^ Knowledge ^¤^ Reinforcement^¤^ Social influences^¤^ Social influences^#^ Social/professional role & identity^¤^ Environmental context & resources^#^^.^ Social influences^#^ Feedback processes^#^ Subjective norms^¤^ Behavior regulation^#^ Feedback processes^#^ Behavioral regulation^¤^
5	*Motivational interviewing:* The health professionals were trained in using attitudes and conversational techniques from motivational interviewing ^2^ in communication with patients during goal setting and follow-up calls. To guide the health professionals, a booklet was used, with examples and suggestions for dialogue and collaboration, including checklists and scales for self-monitoring of therapeutic skills. Using motivational interviewing included: Provide empathy. Seek collaboration. Emphasize autonomy. Affirm patients’ strengths, efforts, intentions, and worth. Give information. Examine and resolve ambivalence related to health behavior change. Promote “change talk.”	Social support (general) [3.1] Social support (emotional) [3.3] Valued self-identity/ Self-affirmation [13.4] Information about health consequences [5.1] Pros and cons [9.2]	Environmental context & resources^#^ Social influence^#^ Intention^¤^ Knowledge^#^ Optimism^#^ Intention^#^ Attitude towards the behavior^#^ Beliefs about consequences^#^ Attitude towards the behavior^#^ Motivation^#^
	*Motivational interviewing* Weaken “sustain talk.” Provide simple and complex reflections.	Self-talk [15.4] Reframing [13.2] Reframing [13.2] Cognitive dissonance [13.3] Comparative imagining of future outcomes [9.3] Reduce negative emotions (includes stress management) [11.2] Focus on the past success [15.3] Feedback on behavior [2.2] Feedback on outcome(s) of behavior [2.2]	General attitudes/beliefs^#^ Feedback processes^¤^ Motivation^#^ Attitude towards the behavior^#^ Motivation^#^ Self-image^¤^ Emotion^¤^ General attitudes/beliefs^¤^ Attitude toward the behavior^¤^ Beliefs about consequences^#^ Emotion^#^ Behavioral regulation^#^ Beliefs about capabilities^¤^ Beliefs about capabilities^#^ Needs ^¤^ Motivation^#^ Feedback processes^#^ Reinforcement ^¤^

Behavior Change Technique (BCT): the active ingredients expected to bring about behavioral change (e.g. feedback on behavior). Mechanisms of Action (MoA): the processes through which a behavior change technique affects behavior (e.g. behavioral regulation). BCTs are defined according to the terminology used in

*The Behavior Change Technique Taxonomy (v1)^3^, [numbers in brackets] are taxonomy identifiers.

MoA according to **The Theory and Techniques Tool developed by The Human Behaviour Change (HBC) project: https://theoryandtechniquetool.humanbehaviourchange.org/tool.

^Ω^ Study materials in Norwegian are available online: https://diakonhjemmetsykehus.no/nkrr/prosjekter/bridge-studien#materiale-fra-bridge-studien.

§ SMART: Specific, measurable, achievable, realistic/relevant, and timed4.

β Tutorial on goal setting: https://www.youtube.com/watch?v=h1O4nj3t_DA&t=8s

¶ PSFS: Patient Specific Functional Scale.

# Link (¤ marginal/inconclusive link) between BCT and MoA according to criteria set by the HBC project.

¤ Inconclusive link: inconsistent or marginal links between BCT and MoA.

1. Scobbie L, McLean D, Dixon D, et al. Implementing a framework for goal setting in community-based stroke rehabilitation: a process evaluation. *BMC health services research* 2013; 13: 190. 2013/05/28. DOI: 10.1186/1472-6963-13-190.

2. Moyers TB, Rowell LN, Manuel JK, et al. The Motivational Interviewing Treatment Integrity Code (MITI 4): Rationale, Preliminary Reliability and Validity. *Journal of substance abuse treatment* 2016; 65: 36–42. 2016/02/15. DOI: 10.1016/j.jsat.2016.01.001.

3. Michie S, Richardson M, Johnston M, et al. The Behavior Change Technique Taxonomy (v1) of 93 Hierarchically Clustered Techniques: Building an International Consensus for the Reporting of Behavior Change Interventions. *Annals of Behavioral Medicine* 2013; 46: 81–95. DOI: 10.1007/s12160-013-9486-6.

4. Bovend’Eerdt TJ, Botell RE and Wade DT. Writing SMART rehabilitation goals and achieving goal attainment scaling: a practical guide. *Clin Rehabil* 2009; 23: 352–361. 2009/02/25. DOI: 10.1177/0269215508101741.

**Table A2.** The BRIDGE protocol Fidelity Checklist and method used to define per-protocol population.

**Table 1. Fidelity Checklist (CL)** for delivery of the BRIDGE-intervention operationalized in 18 subcomponents (CL variables), completed by the patients’ primary contact at the rehabilitation center. Presented with a description of the content of the subcomponents and keys for variable coding, as it was used to define the per-protocol population (PPP).						
Delivered “Yes”	Delivered “No”		Not applicable		Missing	
Coded as 1	Coded as 0		Coded as 3		Coded as .	
P = the patient. The team = the different health personnel involved at the rehabilitation center						
**Initial goal process and collaboration to defining rehabilitation goals**					**CL variable**	**Enter code**
I have provided P with a “Workbook for use during the rehabilitation stay” and asked him/her to prepare for the goal-setting process by reading this booklet, and by viewing the film about goal setting on www.youtube, and by doing the coloring exercise “Where does the shoe pressure?” (mapping problematic life areas according to a traffic-light principle).					**CL1**	
I have/the team has collaborated with P on the development of rehabilitation goals and P has written 3–5 goals in his/her workbook.					**CL2**	
I have introduced P to the electronic portal and P has logged in with his/her secure credential.					**CL3**	
P has recorded his/her goals in the electronic portal (in the Patient Specific Functional Scale) and also completed the other questionnaires in the portal on admission to rehabilitation.					**CL4**	
**Rehabilitation plan and implementation phase**						
For each goal, P has in collaboration with me/the team developed a rehabilitation plan in his workbook. The plan includes strategies for resolving potential risk situations and implementation barriers.					**CL5**	
I have/the team has given P positive feedback on what has worked well in the implementation of planned measures.					**CL6**	
P and I/the team have over time adjusted the specific measures and sub-goals according to the patient's needs, in order to achieve a belief in mastery (self-efficacy) and agreed on expectations of the result.					**CL7**	
**Rehabilitation goals after discharge and the individually tailored follow-up plan**						
P has written 3–5 goals in the workbook for the time after discharge and P has transferred these goals to the electronic portal (Patient Specific Functional Scale at discharge).					**CL8**	
P has completed the rehabilitation plans for each of the goals, including strategies for resolving potential risk situations and barriers. P has put this in writing in the last part of his/her workbook.					**CL9**	
P has a distinct plan for his/her own efforts and has transferred it as “plans independent of health personnel” in the electronic portal.					**CL10**	
I have /the team has collaborated with P about relevant follow-up interventions from other services, and P has entered the result of this collaboration in the electronic portal as “follow-up in the municipal health service after the rehabilitation discharge.”					**CL11**	
P and I have agreed on a date and time for a follow-up phone call concerning rehabilitation goal progression and goal-directed activities at home, approximately one month after discharge.					**CL12**	
**Use of the electronic portal and the digital feedback report after discharge**						
I am assured that P feels informed and capable to log in to the electronic portal at home.					**CL13**	
I have provided P with information on how the digital feedback report (regarding P's scores on the rehabilitation core set) can be used after discharge, alone or together with others, as a support to achieve the goals and maintain the initiated activities and lifestyle changes.					**CL14**	
**Follow-up after discharge**						
I have conducted the first mandatory phone call guidance with P, as agreed.					**CL15**	
If applicable: several additional phone call follow-ups have been implemented (2, or 3, or up to maximum of 4 calls).					**CL16**	
Guidance on goal-directed activities and goal attainment have been topics in the telephone conversation(s), including an evaluation of potential needs for necessary follow-up in the municipality or local community.					**CL17**	
If needed: I have helped P to contact relevant healthcare services after discharge.					**CL18**	
**CL sum (counts of yes) ≥ 16 was required to be included in the PPP**						
**Table 2. Information from the electronic portal (EP) reported by the patients in the BRIDGE intervention group, was utilized to construct a variable 19 containing information on follow-up after discharge, which again was used to define the per-protocol population (PPP).**						
	**Information**	PP	PP	Not PP	Not PP	Not PP
A	P has stated a need for follow-up after discharge	Yes = 1	No = 0	Yes = 1	Yes = 1	Yes = 1
B	P and the team have planned a follow-up after the discharge	Yes = 1	–	Yes = 1	No = 0	No = 0
C	P has received at least one type of follow-up in his/her residential municipality (out of 13 predefined options) according to P's defined need(s) at rehabilitation discharge, within 7 months after discharge	Yes = 1	–	No = 0	Yes = 1	No = 0
**EP variable 19 (yes/no):** **a “yes” was required to be included in the PPP**		Yes, included	Yes, included	No, not included	No, not included	No, not included

^1−6^ **The per-protocol population in the BRIDGE trial was based on information from two sources:** A fidelity checklist for the delivery of 18 intervention sub-components, completed by the patient's primary healthcare contact at the rehabilitation centers during the intervention delivery (Table 1, items 1–18) (yes/no). An electronic portal in which the patients in the intervention group recorded from their home settings whether needed and planned follow-up was received, or not, after rehabilitation discharge (Table 2, item 19) (yes/no). **To be included in the PP population, the patients had to:** Either A) have reported a need for follow-up after rehabilitation discharge, B) planned follow-up according to needs before discharge, and C) received at least one type of follow-up in his/her residential municipality according to stated needs, within 7 months after rehabilitation discharge, or reported no need for follow-up after discharge (Table 2). The rationale behind the construction of the composite variable 19 (yes/no) is shown in Table 2, and a final “yes” was regarded as mandatory to be included in the per-protocol population. In addition, the patients had to have contributed with data on the primary outcome at the 7 months measurement time point. Furthermore, 16 out of the 18 checklists (CL) items had to be verified delivered (ticked yes) by the patient’s primary contact (health professional) at the rehabilitation center.

**Table A3.** Listed reasons for patient rejection of study participation, exclusion, and drop out.

**T0 reasons for declining participation (total n = 348)^1^**	
**in the control phase (n = 166)**	**in the intervention phase (n = 182)**
Did not fulfill inclusion criteria (n = 46) Concerns regarding electronic data security (n = 1) Lack of computer skills or not fond of electronic tasks (n = 7) Not interested or no stated reason (n = 63) Lack of energy or questionnaires and/or intervention perceived as too comprehensive (n = 24) Reluctance towards longstanding commitment or a follow-up period decided by others (n = 13) Other illness (n = 2) Other reasons (n = 10)	Did not fulfill inclusion criteria (n = 24) Lack of computer skills or not fond of electronic tasks (n = 5) Not interested or no stated reason (n = 125) Lack of energy or questionnaires and/or intervention perceived as too comprehensive (n = 16) Reluctance towards longstanding commitment or a follow-up period decided by others (n = 10) Other illness (n = 2)
**T1 reasons for exclusion or withdrawal before baseline assessments (total n = 46)^2^**	
**in the control phase (n = 26)**	**in the intervention phase (n = 20)**
Incomplete baseline data (n = 4) Did not fulfill inclusion criteria (n = 6) Technical login problems (n = 7) Concerns regarding electronic data security (n = 1) Not interested or no stated reason (n = 3) Lack of energy or questionnaires and/or intervention perceived as too comprehensive (n = 3) Other illness (n = 2)	Incomplete baseline data (n = 3) Did not fulfill inclusion criteria (n = 2) Technical login problems (n = 3) Lack of computer skills or not fond of electronic tasks (n = 1) Not interested or no stated reason (n = 4) Lack of energy or questionnaires and/or intervention perceived as too comprehensive (n = 7)
**T2 reasons for exclusion or withdrawal before discharge (total n = 23)^3^**	
**in the control phase (n = 11)**	**in the intervention phase (n = 12)**
Not interested or no stated reason (n = 5) Lack of energy or questionnaires and/or intervention perceived as too comprehensive (n = 2) Other illness (n = 4)	Interrupted rehabilitation before completing discharge assessments (n = 1) Concerns regarding electronic data security (n = 1) Not interested or no stated reason (n = 3) Lack of energy or questionnaires and/or intervention perceived as too comprehensive (n = 5) Other illness (n = 2)
**T3 reasons for exclusion or withdrawal before 2 months follow-up (total n = 32)^4^**	
**in the control phase (n = 16)**	**in the intervention phase (n = 16)**
Not interested or no stated reason (n = 13) Lack of energy or questionnaires and/or intervention perceived as too comprehensive (n = 2) Other illness (n = 1)	Technical login problems (n = 1) Not interested or no stated reason (n = 11) Lack of energy or questionnaires and/or intervention perceived as too comprehensive (n = 2) Deterioration of health status (RMDs) (n = 2)
**T4 reasons for exclusion or withdrawal before 7 months follow-up (total n = 30)^5^**	
**in the control phase (n = 13)**	**in the intervention phase (n = 17)**
Lack of computer skills (n = 1) Not interested or no stated reason (n = 9) Lack of energy or questionnaires and/or intervention perceived as too comprehensive (n = 1) Deterioration of health status (RMDs) (n = 1) Other illness (n = 1)	Technical login problems (n = 1) Not interested or no stated reason (n = 15) Other illness (n = 1)
**T5 reasons for exclusion or withdrawal before 12 months follow-up (total n = 32)^6^**	
**in the control phase (n = 20)**	**in the intervention phase (n = 12)**
Technical login problems (n = 2) Not interested or no stated reason (n = 18)	Technical login problems (n = 2) Not interested or no stated reason (n = 9) Other reasons (n = 1)

^1−6^ T0 = request for participation, T1 = baseline, T2 = discharge, T3 = two months follow-up, T4 = seven months follow-up, T5 = twelve months follow-up.
